# Increase in stroke risk in patients with head and neck cancer: a retrospective cohort study

**DOI:** 10.1038/bjc.2011.361

**Published:** 2011-09-13

**Authors:** C-N Chu, S-W Chen, L-Y Bai, C-H Mou, C Y Hsu, F-C Sung

**Affiliations:** 1 Department of Radiation Oncology, China Medical University Hospital and College of Medicine, 2 Yude Road, Taichung 404, Taiwan; 2 Division of Hematology and Oncology, China Medical University Hospital and College of Medicine, 2 Yude Road, Taichung 404, Taiwan; 3 Management Office for Health Data, China Medical University Hospital and College of Medicine, 2 Yude Road, Taichung 404, Taiwan; 4 Graduate Institute of Clinical Medical Science, China Medical University, 91 Hsueh-Shih Road, Taichung 404, Taiwan; 5 Department of Public Health, China Medical University, 91 Hsueh-Shih Road, Taichung 404, Taiwan

**Keywords:** epidemiology, head and neck cancers, incidence, population-based study, stroke

## Abstract

**Background:**

This study investigated the stroke risk in patients with head and neck cancers (HNCs) using population-based data.

**Methods:**

From claims collected in the Taiwan National Health Insurance database, we identified 13 390 HNC patients with diagnosis made in 2000–2002. A reference cohort of 53 517 non-cancer individuals matched for age, gender, and stroke risk factors was used for assessing stroke risk in follow-up to 2008.

**Results:**

The overall stroke incidence was 1.44-fold higher in the HNC than in the reference cohort (11.4 *vs* 7.9 per 1000 person-years). Adjusted hazard ratios (HRs) were 1.54 (95% confidence interval (CI): 1.40–1.68) for ischaemic stroke and 1.36 (95% CI: 1.09–1.69) for haemorrhagic stroke. The cancer-to-reference stroke incidence rate ratio was age dependent and the highest in the age group younger than 40 years (5.45, 95% CI: 3.78–7.87) and decreased with aging. Comparing different therapeutic modalities, HNC patients receiving both radiotherapy (RT) and chemotherapy (CT) had the highest stroke risk (HR: 1.46, 95% CI: 1.22–1.74), followed in sequence by those who had CT alone, RT alone, and without therapy.

**Conclusion:**

Patients with HNC are at increased risk of developing stroke, especially in the young age group and in those who received both RT and CT.

Cancer is the leading cause of death in developed as well as developing countries. Stroke, a common neurological complication of cancer (Lindvig *et al*, 1990), adds burden to patients who suffer from cancer. The occurrence of stroke in cancer patients has been ascribed to many risk factors, including tumour-related disorders, coagulation disturbances, infection, complications of cancer therapies, and paraneoplastic causes (Lindvig *et al*, 1990; Rogers, 2003; Cestari *et al*, 2004; Nguyen and DeAngelis, 2006; Zhang *et al*, 2006; Grisold *et al*, 2009). An example of cancer as a stroke risk factor is radiation therapy (RT) for head and neck cancers (HNC) that accelerates carotid artery athrosclerosis resulting in stroke as a late complication (Rogers, 2003).

In the recent US cancer statistics, HNC acounted for ∼3% of adult malignancies (Jemal *et al*, 2006). In North America and Europe, HNC usually arises from the oral cavity, oropharynx, or larynx, whereas nasopharyngeal cancer is more common in Mediterranean countries and Southeast China. The incidence of oral and pharyngeal (including oral cavity, oropharynx, and hypopharynx) cancer has increased rapidly in Asia and the South Pacific area, including Taiwan (Chiba, 2001). HNC is strongly associated with certain environmental and lifestyle risk factors, including smoking, alcohol consumption, and certain strains of viruses, such as human papillomavirus (Freedman *et al*, 2007; Feng *et al*, 2009; Ridge *et al*, 2009). Head and neck cancer is highly curable if detected early, usually with surgery. In addition, RT and chemotherapy (CT) may also be of benefit. Head and neck cancer survivors may have compromised quality of life because of treatment-related complications even after successful definitive therapy. One of the adverse events of HNC is stroke. However, systematic assessment of stroke risk among HNC patients has not been made.

Because the risk of stroke increases with age (Hollander *et al*, 2003), age-specific and age-adjusted analyses are required in assessing cancer-related stroke risk. To the best of our knowledge, no study has investigated the risk of stroke in HNC patients using a representative sample of adequate size, as well as taking into consideration comorbidity and age. Furthermore, a long-term follow-up study is essential because of the late occurrence of treatment-related complications. Whether the stroke risk profile in patients with HNC is different from those without HNC remain to be determined. Therapy-related stroke can occur in all three modalities: surgery, RT, and CT. In HNC patients, RT alone or RT combined with CT is the mainstay for definitive or adjuvant treatment. Previous studies have shown that the stroke risk increases among subjects receiving RT (Dorresteijn *et al*, 2002; Haynes *et al*, 2002; Bowers *et al*, 2005; Moser *et al*, 2006; Scott *et al*, 2009). However, except for one study that used a sibling control (Bowers *et al*, 2005), most studies did not establish the independent contribution of RT to stroke by comparing HNC patients with and without RT.

In this study, we hypothesise that HNC patients exhibit increased risk of stroke compared with the general population. The Taiwan National Health Insurance (NHI) database was used to conduct this population-based study. With a large study sample size, the study subjects can be stratified by demographic factors, comorbidity, and therapeutic modalities. Comparison was made with a reference cohort matched for age, gender, and comorbidity, and free from any cancer.

## Materials and Methods

### Data source and study subjects

The Taiwan NHI is a universal health insurance system started in March 1995 by the Taiwan Department of Health after integrating 13 insurance programs. Approximately 99% of the population has been covered in this system since 1999. The National Health Research Institute (NHRI) has computerised medical claims and selected sets of healthcare data for administrative use and research, as described in a large retrospective study (Lin *et al*, 2008). We used 2000–2008 NHRI data, which include all inpatient and ambulatory care records for cancer care, registry for catastrophic illnesses, and basic demographic information. We applied codes of the International Classification of Diseases, 9th Revision, Clinical Modification (ICD-9-CM) and A-code to retrieve information on diagnosis.

Based on the registry for catastrophic illnesses, we identified 13 390 cases of HNC (ICD-9-CM code 140-149 and A-code A080), newly diagnosed in 2000–2002, as the study cohort. Patients with prior stroke or incomplete information were excluded. Head and neck cancer includes malignant neoplasms of tongue (17.5%), oral cavity and oropharynx (33.5%), nasopharynx (29.6%), hypopharynx (8.1%), and others (11.3% including lip, gum, floor of mouth, salivary gland, and ill-defined sites). The date the diagnosis of HNC had been made was set as the index date for beginning the measurement of follow-up person-years. From the reimbursement claim data files, using a ratio of 1 : 4, 53 517 individuals free from any cancer and prior stroke and matched for gender, age, comorbidity (hypertension, diabetes mellitus, or both), and index dates, were randomly selected as the reference cohort. The confirmation of stroke events was based on inpatient records in the NHI database.

### Statistical analysis

The retrieved patient information also includes selected sociodemographic characteristics, such as gender, date of birth, residential area, occupation, and clinical features including comorbidity. Comorbidity retrieved for the present study was restricted to two key stroke risk factors, hypertension (ICD-9-CM 401-405, as well as A-code A260 and A269), diabetes mellitus (ICD-9-CM 250 and A-code A181), or both. Each subject was followed from the index date to the occurrence of stroke (ICD-9-CM 433-438 or A-code A292-294 and A299), death, loss to follow-up, withdrawal from the insurance policy, or until 31 December 2008 with follow-up person-years estimated. We first calculated the incidence densities of stroke in follow-up for both the study and reference cohorts.

Distributions of gender, age (<40, 40–49, 50–59, and ⩾60 years), and comorbidity were compared between the study and reference cohorts, and examined using the *χ*^2^-test. The gender-, age-, and comorbidity-specific incidence densities of stroke were also measured for both cohorts. To assess the relative risk in each subgroup between the two cohorts, we measured the ratios of incidence rate. Cox proportional hazard regression analysis was used to estimate the stroke risks associated with HNC. Hazard ratios (HRs) and 95% confidence intervals (CIs) were provided in the Cox models. We also used the Kaplan–Meier survival analysis to estimate proportions of the studied subjects who did not suffer from stroke during the follow-up period in both cohorts.

To assess the effect of therapy, the study cohort was divided into four subgroups based on therapeutic modalities: RT only; CT only; combination of RT and CT (RT/CT); and neither RT nor CT (non-RT/CT). Because of the limitation in precisely categorising the extent of surgery using the ICD codes from the NHRI records, no separate group receiving surgery only was identified. Using the non-RT/CT group for comparison, we examined whether RT, CT, or RT/CT was a stroke risk factor. HRs of stroke in the RT, CT, and RT/CT groups in reference to the non-RT/CT group were calculated. All data analyses were performed using the SAS 9.1 statistical package (SAS Institute Inc., Cary, NC, USA).

## Results

The HNC and reference cohorts were similar in distributions of gender, age, and comorbidity, with the median age of 50.1 years (Table 1). The dominant gender was male (85.0%). Both cohorts had the same prevalence rates of diabetes mellitus (13.0%), hypertension (16.1%), or both (5.4%).

**Figure 1:**
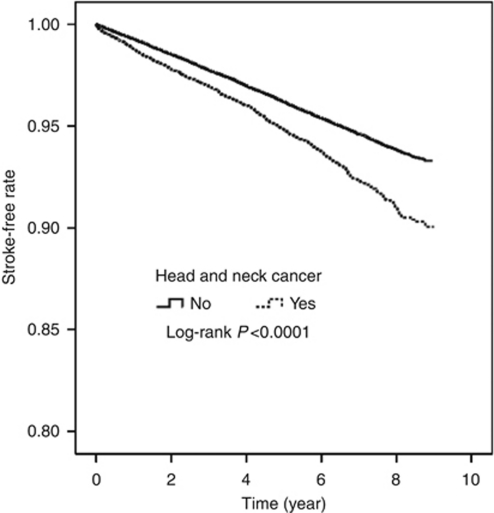
Kaplan–Meier analysis comparing proportions of stroke between patients with head and neck cancers and reference cohort (the time axis was calculated from the index date).

After 5–7 years of follow-up, this study identified 694 cases of stroke among patients with HNC and 2915 in the reference cohort (Table 2). The overall incidence of stroke was 1.44-fold higher in the HNC than the reference cohort (11.4 *vs* 7.9 per 1000 person-years) with an adjusted HR of 1.52 (95% CI: 1.40–1.65). Generally, the incidence of stroke increased with age in both cohorts. However, the age-specific HNC-to-reference incidence rate ratio decreased with aging, with the highest noted in those HNC patients younger than 40 years (HR: 5.76, 95% CI: 3.99–8.33). The comorbidity-specific HRs show that hypertension and diabetes mellitus were, as predicted, significant stroke risk factors (HR: 1.37 *vs* 1.68, both with *P*<0.0001). Data analysis also estimated the incidence by the follow-up year. With follow-up time beginning from years 2 to 4, the HR increased to the highest level of 1.97 (95% CI: 1.61–2.41) in years 7–8 after controlling for gender, age, hypertension, and diabetes mellitus. The stroke-free curves are depicted in Figure 1. Stroke appeared to occur immediately in small proportions in the HNC cohort, but a growing trend of disparity was observed between the two cohorts when the follow-up duration increased (*P*<0.0001).

Table 3 shows that the incidence of ischaemic stroke was approximately 6.1-fold higher than that of haemorrhagic stroke among patients with HNC. The corresponding incidence figure was 5.4 in the reference cohort. Compared with normal individuals, the adjusted HR was slightly higher for ischaemic stroke (1.54, 95% CI: 1.40–1.68) than haemorrhagic stroke (1.36, 95% CI: 1.09–1.69), but the difference was not significant.

Table 4 shows the stroke risk at each of the five HNC sites. Patients with nasophrynx had a trend of higher risk for ischaemic stroke with a HR of 2.61 (95% CI: 2.29–2.97), whereas hypopharynx had higher risk for haemorrhagic stroke (HR: 1.96, 95% CI: 1.01–3.79). Table 5 lists the stroke risk associated with treatment modalities. Compared with the patients with neither RT nor CT, the risk of stroke was the highest for the cancer patients receiving both RT and CT (HR: 1.46, 95% CI: 1.22–1.74), followed in sequence by those who had only CT, only RT, and neither CT nor RT.

## Discussion

This study is the first population-based cohort analysis of the stroke risk in HNC patients, compared with a reference group free of cancer and matched for age, gender, and comorbidity. Results show that HNC increased the risk of stroke with a trend for more ischaemic than haemorrhagic stroke. As shown in Figure 1, disparity in stroke-free rate grew with extension of the follow-up period, suggesting delayed impact of HNC and therapies on the stroke risk. Treatment-related late cerebrovascular damage turned out to be prominent when more patients survived for longer periods. Furthermore, patients with nasopharyngeal carcinoma showed a trend for higher risk for ischaemic stroke, whereas hypopharynx had a higher tendency for haemorrhagic stroke.

It is worth noting that the stroke risk in HNC patients was relatively higher in patients of younger age. This important finding is made on the well-known observation that the stroke risk increases with aging as clearly shown in Table 2 and reported by others (Chen *et al*, 2008; Lip and Halperin, 2010). This is likely related to the notion that in HNC patients of older age, the impact of established stroke risk factors carries greater weight in the pathogenesis of stroke. A similar trend of relatively higher vulnerability for stroke in younger patients was observed in a study on the long-term risk of cerebrovascular disease associated with the use of RT and CT in survivors of Hodgkin’s lymphoma (De Bruin *et al*, 2009). Because of recent growing trends of HNC occurrence in younger patients (Ridge *et al*, 2009), it is prudent to include stroke prevention measures in follow-up of HNC survivors, especially for those of younger age. Results on comorbidity disclosed that stroke risk is increased further in patients with hypertension, diabetes, or both. Thus, close surveillance for HNC patients with hypertension, diabetes, or both for more intense management of these stroke risk factors is needed.

According to previous studies, the most frequent cancer types associated with stroke are urogenital, breast, gastrointestinal, haematological, and lung cancers (Cestari *et al*, 2004; Grisold *et al*, 2009; Stefan *et al*, 2009). In addition, the frequency of ischaemic stroke exceeded that of haemorrhagic stroke in cancer patients, a situation similar to that in a non-cancer population. Except for deep vein thrombosis, other stroke-related risk factors do not significantly vary between cancer and non-cancer patients (Rogers, 2003; Stefan *et al*, 2009). To the best of our knowledge, no specific malignancy that causes stroke more often than cancer types has been identified (Li *et al*, 2006; Stefan *et al*, 2009).

Previous studies have reported various risk levels for stroke for HNC patients receiving RT. The risk may vary with the severity of internal carotid artherosclerosis. The frequency of internal carotid stenosis following neck RT ranged from 12 to 60% (Cheng *et al*, 1999), although controversies remain (Marcel *et al*, 2005). The stroke risk associated with RT to the neck appears variable. In addition, different study designs also contributed to risk variation (Dorresteijn *et al*, 2002; Haynes *et al*, 2002; Hoffman *et al*, 2006; Moser *et al*, 2006; Scott *et al*, 2009). In a systematic review, Scott *et al* (2009) reported a range of 2.1–8.5-fold higher.

In the present study, we note a higher trend for ischaemic stroke for patients with nasopharyngeal cancer. This finding raises the concern of therapy-related complications. In definitive RT or concurrent chemoradiotherapy (CCRT) for HNC patients, the prescribed RT dosage is not particularly high for nasopharyngeal cancer patients. However, many patients with HNC, except nasopharyngeal cancer, may receive adjuvant RT/CCRT following surgery, in which the RT dose is lower. Thus, there is a need to analyse nasopharyngeal cancer populations using similar stratification strategies to precisely examine stroke risk associated with cancer *per se* or therapies, particularly treatment with both CT and RT.

Results presented here should be interpreted with caution because of the following limitations. First, smoking and alcohol consumption are two well-known lifestyle risk factors of HNC. The prevalence of smoking and drinking was likely higher in the study cohort than in the reference cohort. Information on smoking was unavailable from the insurance claims. However, diagnosis for alcoholism, which indicates heavy drinking, was available from the claims. Smoking generally parallels drinking behaviour. Further data analysis shows a higher prevalence of alcoholism in the HNC cohort than in the reference cohort (2.02 *vs* 0.89%). Alcoholism was more prevalent in patients with malignant neoplasm of the hypopharynx (5.84%) than other types of HNC in the present study. It is well known that excessive alcohol intake is associated with increased risk of haemorrhagic stroke (Rymer, 2011). Second, the NHRI records do not provide information on treatment dosage or intensity. Finally, patients with HNC who received surgery alone could not be specified because of the inherent shortcoming of the NHI database.

## Conclusion

In this retrospective cohort study carried out with large samples, patients with HNC had 52% excess risk of developing stroke compared with a reference population free of cancer and matched for age, gender, and stroke risk factors including hypertension, diabetes, or both. We also note that the stroke risk for HNC patients was age dependent with the highest in those younger than 40 years. The stroke risk was also higher among those receiving both RT and CT. Age and treatment modality are important factors for consideration in preventing stroke in patients with HNC.

## Figures and Tables

**Table 1:** Distributions of gender, age, and comorbidity in patients with head and neck cancers and reference cohort identified in 2000–2002

		**Head and neck cancer**		
	**Total, *N*=66 907**	**No, *N*=53 517**	**Yes, *N*=13 390**	
	***n* (%)**	***n* (%)**	***n* (%)**	***P*-value**
*Gender*				0.98
Female	10 038 (15.0)	8030 (15.0)	2008 (15.0)	
Male	56 869 (85.0)	45 487 (85.0)	11 382 (85.0)	
				
*Age (years)*				1.00
<40	11 410 (17.1)	9127 (17.1)	2283 (17.1)	
40–49	21 739 (32.5)	17 387 (32.5)	4352 (32.5)	
50–59	16 872 (25.2)	13 495 (25.2)	3377 (25.2)	
⩾60	16 886 (25.2)	13 508 (25.2)	3378 (25.2)	
Median±s.d.^a^	50.1±13.2	50.1±13.2	50.1±12.8	
				
*Comorbidity*				
Hypertension	10 779 (16.1)	8621 (16.1)	2158 (16.1)	0.98
Diabetes mellitus	8727 (13.0)	6973 (13.0)	1754 (13.1)	0.83
Both	3604 (5.39)	2881 (5.38)	723 (5.40)	0.94

*χ*^2^-test was used for categorical data.

a Brown-Mood test for medians.

**Table 2:** Incidence and hazard ratio of stroke by demographic factor and comorbidity among patients with head and neck cancers and reference cohort

	**Head and neck cancer**									
	**No**			**Yes**					**Adjusted^a^**	
	**Cases**	**Person-years**	**Rate**	**Cases**	**Person-years**	**Rate**	**Rate Ratio**	**(95% CI)**	**HR**	**(95% CI)**
Overall	2915	369 020	7.90	694	60 983	11.4	1.44	(1.33–1.57)^***^	1.52	(1.40–1.65)^***^
										
*Gender*										
Female	435	65 138	6.68	110	10 783	10.2	1.52	(1.07–1.62)^*^	1.53	(1.24–1.88)^***^
Male	2480	312 882	7.93	584	50 200	11.6	1.47	(1.34–1.61)^***^	1.57	(1.43–1.72)^***^
										
*Age (years)*										
<40	57	65 882	0.87	57	12 085	4.72	5.45	(3.78–7.87)^***^	5.76	(3.99–8.33)^***^
40–49	392	124 335	3.15	146	20 644	7.07	2.24	(1.85–2.71)^***^	2.30	(1.90–2.78)^***^
50–59	689	94 618	7.28	203	15 433	13.2	1.81	(1.54–2.11)^***^	1.84	(1.57–2.15)^***^
⩾60	1777	84 185	21.1	288	12 821	22.5	1.06	(0.94–1.21)	1.06	(0.93–1.20)
										
*Comorbidity*										
Hypertension	928	56 360	16.5	206	9959	20.7	1.26	(1.08–1.46)^*^	1.37	(1.18–1.59)^***^
Diabetes mellitus	752	44 543	16.9	148	7382	20.1	1.19	(1.00–1.42)	1.68	(1.52–1.84)^***^
Both	374	18 359	20.4	81	3239	25.0	1.23	(1.00–1.62)	1.32	(1.04–1.68)^*^
										
*Follow-up (years)*										
<2	773	104 826	7.37	251	22 033	11.4	1.54	(1.36–1.78)^***^	1.64	(1.42–1.90)^***^
2–4	798	100 467	7.94	150	16 356	9.18	1.16	(0.97–1.38)	1.26	(1.05–1.50)^*^
5–6	795	96 087	8.27	164	13 847	11.8	1.43	(1.21–1.70)^***^	1.53	(1.29–1.81)^***^
7–8	495	60 391	8.20	118	7911	14.9	1.82	(1.49–2.23)^***^	1.97	(1.61–2.41)^***^
⩾8	54	7248	7.45	11	836	13.2	1.76	(0.92–3.37)	1.97	(1.03–3.78)^***^

Abbreviations: CI=confidence interval; HR=hazard ratio.

Rate: per 1000 person-years; Ratio: rate ratio of study cohort to reference cohort.

^*^P<0.05, ^***^P<0.0001.

a Adjusted HR: multivariable analysis including gender, age, hypertension, diabetes mellitus, and years after index date.

**Table 3:** Comparison of incidence and risk of stroke by subtype and gender between patients with head and neck cancers and reference cohort

	**Head and neck cancer**							
	**No**		**Yes**		**Unadjusted**		**Adjusted^a^**	
	**Case**	**Rate**	**Case**	**Rate**	**HR**	**(95% CI)**	**HR**	**(95% CI)**
*Both genders*								
HS	455	1.23	98	1.61	1.32	(1.06–1.64)^*^	1.36	(1.09–1.69)^*^
IS	2460	6.67	596	9.77	1.47	(1.34–1.60)^***^	1.54	(1.40–1.68)^***^
								
*Women*								
HS	60	0.92	14	1.30	1.23	(0.69–2.20)	1.34	(0.75–2.40)
IS	375	5.76	96	8.90	1.34	(1.07–1.67)^*^	1.49	(1.19–1.86)^**^
								
*Men*								
HS	395	1.26	84	1.67	1.34	(1.06–1.70)^*^	1.36	(1.08–1.73)^*^
IS	2085	6.66	500	9.96	1.49	(1.35–1.65)^***^	1.55	(1.40–1.71)^***^

Abbreviations: CI=confidence interval; HR=hazard ratio; HS=hemorrhagic stroke; IS=ischemic stroke.

Rate: per 1000 person-years.

^*^P<0.05, ^**^P<0.001, ^***^P<0.0001.

a Mutually adjusted for gender, age, hypertension, and diabetes mellitus.

**Table 4:** Incidence of hemorrhagic and ischemic stroke and multivariable Cox model hazard ratios compared with reference cohort by head and neck cancer sites

					**Total**				**HS**				**IS**	
**Cancer site (ICD)**	***N***	**Person-years**	**Case**	**IR**	**HR**	**(95% CI)**	**Case**	**IR**	**HR**	**(95% CI)**	**Case**	**IR**	**HR**	**(95% CI)**
Tongue (141)	2369	10 739	94	1.25	1.25	(1.02–1.54)^*^	15	1.40	1.22	(0.73–2.05)	79	7.36	1.25	(1.00–1.56)
Oral cavity and oropharynx (145,146)	4503	19 203	201	1.31	1.31	(1.13–1.51)^**^	29	1.51	1.20	(0.82–1.75)	172	8.96	1.32	(1.13–1.54)^**^
Nasopharynx (147)	3957	20 581	293	2.47	2.47	(2.19–2.79)^***^	36	1.75	1.74	(1.24–2.45)^*^	257	12.5	2.61	(2.29–2.97)^***^
Hypopharynx (148)	1078	3185	50	1.60	1.60	(1.21–2.12)^**^	9	2.83	1.96	(1.01–3.79)^*^	41	12.9	1.54	(1.13–2.09)^*^
Others	1483	7275	56	0.89	0.89	(0.68–1.16)	9	1.24	0.96	(0.50–1.86)	47	6.46	0.87	(0.65–1.16)

Abbreviations: CI=confidence interval; HR=hazard ratio adjusted for gender, age, hypertension, and diabetes mellitus; HS=hemorrhagic stroke; IR=incidence rate per 1000 person-years; IS=ischemic stroke. ^*^P<0.05, ^**^P<0.001, ^***^P<0.0001.

**Table 5:** Incidence of stroke and hazard ratios among patients with head and neck cancer by type of treatment

**Treatment**	***N***	**Stroke case**	**Person-year**	**Rate^a^**	**HR**	**(95% CI)**
Non-RT/CT	4387	261	23 525	11.1	1.00	(Reference)
RT	2347	135	11 415	11.8	1.10	(0.89–1.35)
CT	1067	65	5782	11.2	1.31	(1.00–1.35)
RT/CT	5049	233	20 261	11.5	1.46	(1.22–1.74)^***^
Control	53 517	2915	369 020	7.90	0.75	(0.66–0.85)^***^

Abbreviations: CI=confidence interval; CT=chemotherapy; HR=hazard ratio; non-RT/CT=neither radiotherapy nor chemotherapy; RT=radiotherapy alone; RT/CT=radiotherapy and chemotherapy.

Adjusted for gender, age, hypertension, and diabetes.

a Per 1000 person-years.

^***^P<0.0001.
